# Seroprevalence of simian immunodeficiency virus in wild and captive born Sykes' monkeys (*Cercopithecus mitis*) in Kenya

**DOI:** 10.1186/1742-4690-1-34

**Published:** 2004-10-28

**Authors:** Brett R Ellis, Elephas Munene, Debra Elliott, James Robinson, Moses G Otsyula, Scott F Michael

**Affiliations:** 1 Department of Tropical Medicine, Tulane University, New Orleans, LA 70112, USA; 2 Institute of Primate Research, P.O Box 24481, Karen, Nairobi, Kenya; 3 Division of Pediatric Infectious Diseases, Tulane University, New Orleans, LA 70112, USA; 4 Biotechnology Program, Florida Gulf Coast University, Fort Myers, FL 33965, USA

## Abstract

**Background:**

The Sykes' monkey and related forms (*Cercopithecus mitis*) make up an abundant, widespread and morphologically diverse species complex in eastern Africa that naturally harbors a distinct simian immunodeficiency virus (SIVsyk). We carried out a retrospective serological survey of SIV infection from both wild and captive Sykes' monkeys from Kenya. We compared two commercially available, cross-reactive ELISA tests using HIV antigens with a novel SIVsyk antigen-specific Western blot assay and analyzed the data by origin, subspecies, age and sex.

**Results:**

The SIVsyk antigen-specific Western blot assay detected more serum samples as positive than either of the cross-reactive ELISA assays. Using this assay, we found that seroprevalence is higher than previously reported, but extremely variable in wild populations (from 0.0 to 90.9%). Females were infected more often than males in both wild and captive populations. Seropositive infants were common. However, no seropositive juveniles were identified.

**Conclusion:**

We have developed a specific and sensitive Western blot assay for anti-SIVsyk antibody detection. Sykes' monkeys are commonly infected with SIVsyk, but with extremely variable prevalence in the wild. Higher infection prevalence in females suggests predominantly sexual transmission. High infection prevalence in infants, but none in juveniles, suggests maternal antibodies, but little or no vertical transmission.

## Background

Both human immunodeficiency virus-1 (HIV-1) and human immunodeficiency virus-2 (HIV-2) have been evolutionarily linked to zoonotic transmissions of naturally occurring simian immunodeficiency viruses (SIVs) from Central African chimpanzees (*Pan troglodytes troglodytes*) and West African sooty mangabey monkeys (*Cercocebus atys*), respectively [1,2]. In addition, the SIVsmm from sooty mangabeys has been transmitted in captivity to Asian macaques (*Macaca mulatta*) and is pathogenic in that species, producing an acquired immunodeficiency syndrome-like condition [3-6]. Evidence of infection by naturally occurring SIVs has been documented in a variety of different non-human African primate species [7-9] and additional types of SIV are likely to exist in other African primates as well [9].

Despite the importance of naturally occurring SIVs as reservoirs for human disease as well as infections in other primate species, few studies have addressed the distribution or prevalence of these viruses in wild host populations [10]. Research in this area has been limited by the fact that many primate species are endangered in the wild and therefore samples used for SIV studies have often been acquired from research centers and zoos, not wild animals. The patterns of transmission in captive animals may have been influenced by altered contacts or behaviors in captivity and may not reflect natural transmission patterns. Furthermore, not all SIVs grow well in cell culture and it has consequently been difficult to generate specific reagents for their study.

The Sykes' monkey (*Cercopithecus mitis*) belongs to an abundant, widespread and morphologically diverse species complex found throughout East Africa [11-13]. Previous investigations have indicated that these monkeys are infected with a unique virus: SIVsyk. Both wild and captive *C. mitis *have previously been shown to have antibodies that cross-reacted with SIVsmm and SIVagm [14]. A virus isolate (SIVsyk173) was made from one SIVsyk infected animal and an infectious clone was produced, sequenced and shown to form a distinct phylogenetic group from other known SIVs [15]. Very recently, several additional, related SIVsyk sequences have also been reported [16]. These viruses apparently do not grow well in human or rhesus macaque PBMCs, but have been shown to replicate to some degree in immortalized human lymphoid cell lines [17]. SIVsyk also does not produce detectable disease in either the natural host or in rhesus macaques [15,17] (personal observation M. G. Otsyula), consistent with other observations that infection of many natural host species with their specific SIV is non-pathogenic [15].

Here we report the results of a retrospective serosurvey for the prevalence of SIVsyk in wild Sykes' monkey populations in comparison to captive animals. We compare the use of two different cross-reactive ELISA assays with a novel SIVsyk-specific Western blot assay based on the isolate SIVsyk173. We provide a breakdown of seroprevalence results between captive and geographically isolated wild populations, subspecies, sexes and age groups. These data support the utility of using an antigen-specific assay rather than a cross-reactive assay and indicate that the prevalence of SIVsyk infection is highly variable in wild populations and higher in adult animals than previously reported.

## Results

### Comparison of SIVsyk Western blot assay to cross-reactive ELISAs

Two hundred and seventy six serum samples from both wild and captive *C. mitis *were screened for the presence of antibodies cross-reactive to HIV-1 and HIV-2 using two commercial ELISA tests. The 276 samples included 100 sequential (duplicate or triplicate) serum samples from the same animals taken at different times. From these sera, the Innotest HIV-1/HIV-2 antibodies test (Innogenetics, Belgium) detected a total of 132 positive samples and 144 negative samples. The Genescreen HIV-1/2 version 2 test (Biorad, Japan). detected 129 positive samples and 147 negative samples. Five samples gave disparate results using these two tests, four of these samples were positive using the Innotest kit, but negative using the Genscreen test and one sample was positive using the Genscreen test, but negative using the Innotest kit.

**Figure 1:**
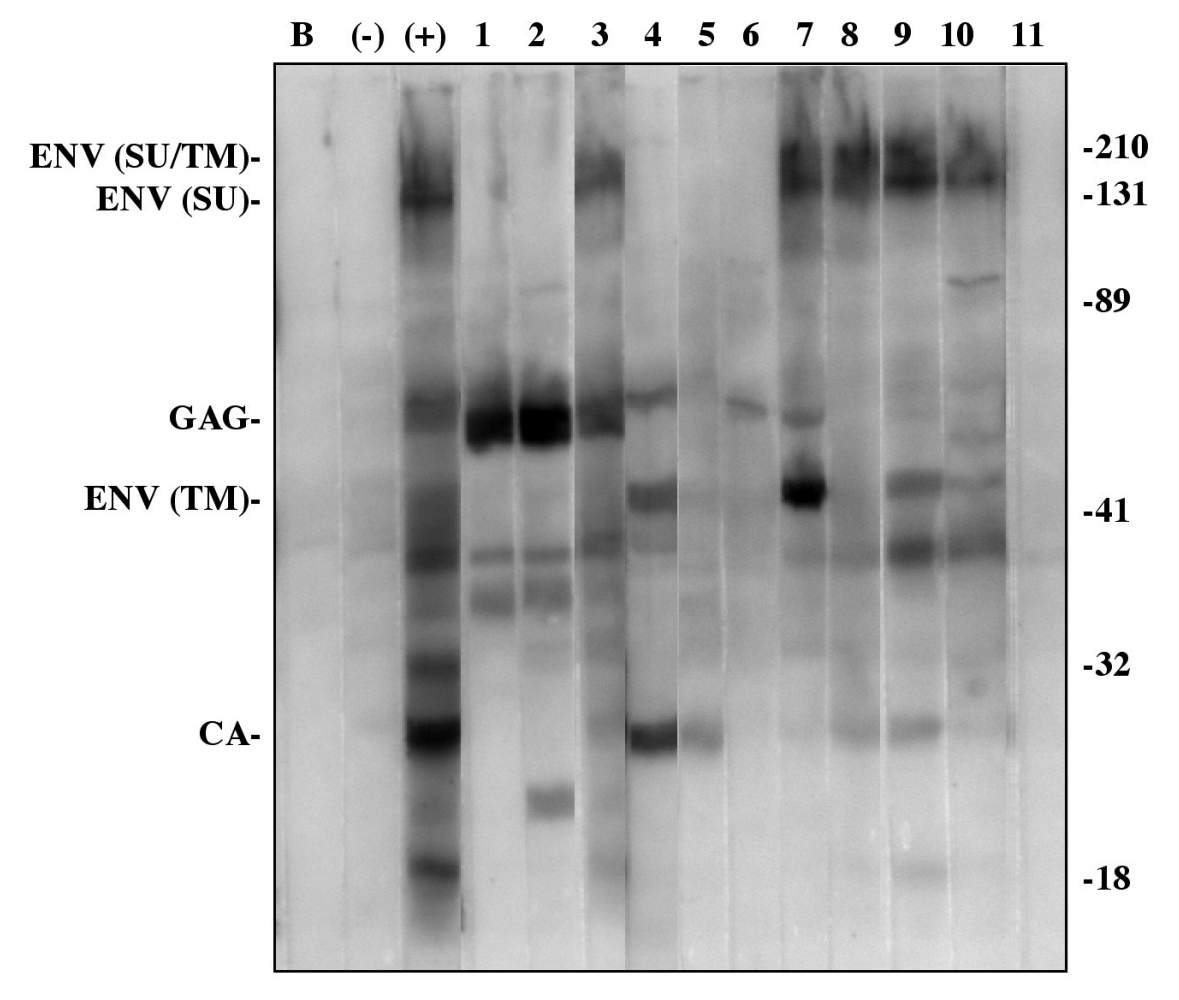
**Representative SIVsyk Western blot results. **Blank (B), Negative serum sample (-), Positive serum sample (+). Lanes 1–10, samples negative by at least one ELISA test, but positive for ENV, GAG or CA by Western. Lane 11, sample positive by both ELISA tests, but negative by Western.

To confirm these results and compare the sensitivity and specificity of these assays, all 276 samples were screened by an SIVsyk viral antigen-specific Western blot assay. Serum samples were considered positive by SIVsyk Western blot assay if they showed reactivity to ENV, GAG or CA proteins. The SIVsyk Western assay identified a total of 138 samples as positive and 138 as negative. Ten samples that were negative by the Genscreen assay and seven samples that were negative by the Innotest assay were identified as positive by the SIVsyk Western assay (Figure 1). One sample that was identified as positive by both ELISA tests was consistently negative by SIVsyk Western. Using the SIVsyk Western assay as the standard, this results in a 99.3% specificity for both ELISA assays and 93.9% and 95.8% sensitivity for the Genscreen and Innotest assays, respectively, for detection of anti-SIVsyk serum antibodies. Using McNemar's repeated measures test, the difference between the Western and the Innotest is just under the 95% confidence limit (p = 0.0703) and the difference between the Western and the Genescreen test is statistically significant (p = 0.0117).

### Serosurvey of wild animals

**Figure 2:**
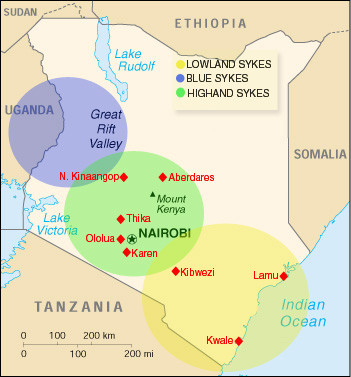
**Map of Kenya, East Africa. **Wild population locations and approximate subspecies ranges are shown.

The results from the SIVsyk Western assay were analyzed according to geographic origin, subspecies, age and sex. Figure 2 shows a map of Kenya indicating the locations of the wild collection sites as well as the approximate ranges of the three Sykes' monkey subspecies. Sequential serum samples were excluded from this analysis, reducing the number of samples considered to 176 (109 wild and 67 captive). Of the 109 individual samples from wild populations, 51 positives were detected, for a total wild seroprevalence of 46.8%. This includes both sexes and all age classes from all locations. Between individual wild populations, the seroprevalence level was quite variable, with a high of 90.9% on the coast at Lamu, to 0% at Kwale, also on the coast. Of wild populations, 25 of 38 adult highland Sykes', 11 of 13 adult lowland Sykes' and 1 of 2 adult blue Sykes' were positive, yielding prevalences of 65.8%, 84.6% and 50.0%, respectively for adults of each subspecies. Among the wild born adult animals, 17 of 26 (65.4%) males and 20 of 27 (74.1%) females were positive. Although 1 of 2 (50.0%) wild born infant males and 1 of 1 (100.0%) wild born infant females were positive, 0 of 3 (0.0%) wild born juvenile males were positive. No wild female juveniles were available for testing. Table 1 provides a list of the seroprevalence for all samples from each wild collection site, as well as a list of prevalence among adults only at each site. Tables 2 and 3 provide a breakdown of wild animal seroprevalence according to subspecies, and age and sex, respectively. A Fisher's exact (2 × 2) contingency test for sex-related differences in the total wild population yielded a p value of 0.559, indicating that sex-related differences in the wild are not statistically significant. An exact 3 × 2 contingency test for age-related differences yielded a p value of 0.069, indicating that age-related differences in the wild population are just slightly not statistically significant at the 95% confidence level. A chi squared 8 × 2 contingency test for differences between the eight wild populations yielded a p value of 0.00099, indicating that the differences between populations was highly significant, although the small size of some of the samples violates the assumptions of this test. Repeating this test using only adult animals is also statistically significant (p = 0.0293), however, the further decrease in sample size using only the data from adult animals further violates the assumptions of this test.

**Table 1:** Anti-SIVsyk serum antibodies among wild Sykes' monkeys according to location. Data for all age groups as well as for adults only in each population is provided. Serum samples from animals with no recorded location are listed as unknown.

**Location**	**Total Positive/Tested**	**Total Percent positive**	**Adults Positive/Tested**	**Adults Percent positive**
Aberdares	5/7	71.4%	4/6	66.7%
Karen	1/4	25.0%	0/3	0.0%
Kibwesi	1/7	14.3%	1/2	50.0%
Kwale	0/9	0.0%	0/1	0.0%
Lamu	10/11	90.9%	9/9	100.0%
N. Kinaangop	3/7	42.9%	0/0	-
Ololua	20/34	58.8%	17/23	73.9%
Thika	3/4	75.0%	3/4	75.0%
Unknown	8/26	30.8%	3/5	60.0%
Total	51/109	46.8%	37/53	69.8%

**Table 2:** Anti-SIVsyk serum antibodies among wild and captive born Sykes' monkeys according to subspecies. Serum samples from wild animals of unrecorded subspecies are listed as unknown. Colony animals of either mixed breeding or unknown origin are listed as unknown.

	**Wild born animals**		**Captive born animals**	
**Subspecies**	**Positive/Tested**	**Percent positive**	**Positive/Tested**	**Percent positive**
Highland	25/38	65.8%	9/17	52.9%
Lowland	11/13	84.6%	3/4	75.0%
Blue	1/2	50.0%	0/0	-
Unknown	14/56	25.0%	12/46	26.1%

Total	51/109	46.8%	24/67	35.8%

**Table 3:** Anti-SIVsyk serum antibodies among wild and captive born Sykes' monkeys according to age and sex. Serum samples from wild animals of undetermined age or sex are listed as unknown.

		**Wild born animals**		**Captive born animals**	
**Age**	**Sex**	**Pos./Tested**	**Percent pos.**	**Pos./Tested**	**Percent pos.**
Adult	Male	17/26	65.4%	4/8	50.0%
	Female	20/27	74.1%	9/13	69.2%
	Subtotal	37/53	69.8%	13/21	61.9%
Juvenile	Male	0/3	0.0%	0/6	0.0%
	Female	0/0	-	0/5	0.0%
	Subtotal	0/3	0.0%	0/11	0.0%
Infant	Male	1/2	50.0%	4/16	25.0%
	Female	1/1	100%	7/19	36.8%
	Subtotal	2/3	66.7%	11/35	31.4%
Unknown	Unknown	12/50	24.0%	0/0	-
Total		51/109	46.8%	24/67	35.8%

### Serosurvey of captive animals

Sixty seven individual serum samples were available from captive animals. The overall seroprevalence in captivity was 35.8% (24/67). This includes both sexes and all age classes. Ten of 17 captive adult highland Sykes' and 3 of 4 captive adult lowland Sykes' were positive, yielding prevalences of 59.0% and 75.0%, respectively for these two subspecies. No known captive Blue Sykes' were available for testing. Among captive adult animals, 4 of 8 (50.0%) males and 9 of 13 (69.2%) females were positive. Four of 16 (25.0%) captive infant males and 7 of 19 (36.8%) captive infant females were positive. However, no captive juveniles (0 of 6 (0.0%) males and 0 of 5 (0.0%) females) were positive. Tables 2 and 3 provide a breakdown of captive animal seroprevalence according to subspecies, and age and sex, respectively. A Fisher's exact (2 × 2) contingency test for sex-related differences in the captive population yielded a p value of 0.646, indicating that sex-related differences in captivity are not statistically significant. An exact 3 × 2 contingency test for age-related differences yielded a p value of 0.001, indicating that age-related differences in captivity are statistically significant.

### Serosurvey comparisons

The overall captive seroprevalence was 35.8%, which is slightly lower than the overall wild seroprevalence of 46.8%. Infection prevalence was highest in adults at 69.8% for wild populations and 61.9% in captive populations. Adult females were infected at a higher prevalence than adult males in both the wild (74.1% (20/27) for wild adult females and 65.4% (17/26) for wild adult males) and captive populations (69.2% (9/13) for captive adult females and 50.0% (4/8) for captive adult males). Infants were also found to have a high seroprevalence at 66.7% (2/3) from wild populations and 31.4% (11/35) from captivity, although few wild infants were surveyed. No seropositive juveniles were identified from either wild (0/3) or captive (0/11) populations. A Fisher's exact (2 × 2) contingency test for sex-related differences in the combined wild and captive populations yielded a p value of 0.455. This indicates that while the there is a clear trend in each data set showing higher prevalence among females, the small numbers of samples do not show a statistically significant difference between infection in males and females. An exact 3 × 2 contingency test for age-related differences in the combined wild and captive populations yielded a p value of 0.00001, indicating that age-related differences in the combined populations are highly statistically significant.

## Discussion

Previous SIV serosurveys of *Cercopithecus mitis *have used assays relying on cross-reactivity between antibodies against SIVsyk and other HIVs or SIVs. To assess the relative effectiveness of the use of cross-reactive tests, we compared serological results using two commercially available HIV-1/HIV-2 ELISA kits with an authentic SIVsyk antigen Western blot assay. The cross-reactive ELISA assays were very specific and in only one out of 276 samples gave a false positive result compared to the SIVsyk antigen Western assay. Curiously, this one particular captive juvenile female was repeatedly positive by both ELISAs but repeatedly negative by Western. This animal may possibly have been infected in captivity with a different SIV that produces cross-reactive antibodies to HIV-1 and HIV-2, but not to SIVsyk. On the other hand, the cross-reactive ELISAs failed to detect between 4.2 to 6.1% of the animals that were positive by the SIVsyk antigen Western assay. This result may be due to increased sensitivity using a Western blot format, but is also possibly due to increased sensitivity to the authentic SIVsyk viral antigens.

Variable SIV infection prevalences have previously been reported in this species. However, information on age, sex and captive or wild status was not provided in any previous survey. Lowenstine et al, 1986 [18] found no evidence of infection in two captive Sykes' monkeys at US zoos using an HIV-1 antigen based ELISA. Ohta, et al, 1988 [19], found 4 out of 18 (22%) Sykes' monkeys from East Africa with evidence of infection, presumably by Western using SIVagm antigen, but no further details were given. Three other reports specifically tested Sykes' monkeys at the Institute for Primate Research in Kenya, but it is unknown which of the samples tested in this survey are from the same animals. Emau, et al, 1991 [17], reported 59 out of 100 (59%) positive using a radioimmuno-precipitation assay cross-reactive with SIVsmm and SIVagm. Tomonaga, et al, 1993 [20], reported 9 out of 35 (25%) lowland Sykes', 13 out of 24 (54%) highland Sykes', and 1 out of 14 (7%) blue Sykes' positive using an immunofluorescence assay with SIVagm antigen. Otsyula et al, 1996 [14], reported 12 out of 35 (34%) positive by Western blot using SIVagm antigen and 8 out of 35 (23%) positive by Western blot using SIVsmm antigen. The low incidence of infection reported by Otsyula, et al 1996 [14] using Western blots with SIVagm as well as SIVsmm antigens supports the hypothesis that the increased sensitivity in the present study is due to the use of SIVsyk antigens and is not likely to be simply due to the use of Western blot assay vs. ELISA.

To avoid age-related bias, a comparison between captive and wild populations should be made using only adult animals because the captive population contained many more samples from infants and juveniles than did the wild population (see Table 3). Both captive and wild adults showed a much higher prevalence than previous reports. Just over 60% of captive adults and nearly 70% of wild adults were seropositive. However, in wild populations, seroprevalence varied greatly according to location (from 0 to 90.9%) and this variability was statistically significant. The differences in seroprevalence may be due to small sample sizes, however, the average troop size in this species is approximately 10 animals [12] and we have sampled between 4 and 34 animals from each individual site (some sites have more than one troop present). Alternatively, host immunological parameters, genetic differences in virus strains, troop size, individual or troop behavior, and habitat may all affect the seroprevalence of these viruses among certain populations.

Although the small sample sizes did not provide statistical significance, in both captive and wild animals, a higher infection prevalence was seen in females than in males. This possibly indicates a predominantly sexual transmission route, rather than transmission by aggressive behavior between males as has been shown in mandrills [21]. Sykes' monkeys usually live in a harem arrangement where a dominant male controls sexual access to the females in the troop. This may explain the variability in seroprevalence among wild populations, since the infection status of a dominant male would heavily influence the status of the entire troop. However, other breeding strategies exist in this species and influxes of multiple solitary males into troops have been documented during periods when multiple females are sexually receptive [22]. Unfortunately, we have no data on the breeding situations in the sampled groups or which samples came from dominant versus lower status males.

In addition to the adults, a high seroprevalence was also observed in infants. However, no seropositive juveniles were identified, even though 14 juvenile animals were sampled from both wild and captive populations. A possible explanation is that infants are seropositive due to the presence of maternal antibodies in breast milk, but little or no actual maternal-fetal transmission occurs either *in utero*, during birth or through breastfeeding. An alternative possibility to consider is that SIVsyk infections could be cleared by infants and that adult seroprevalence is due to later exposure.

## Conclusions

In order to investigate the infection prevalence of SIVsyk in the common and widespread Sykes' monkey in East Africa, we used two commercially available ELISA assays and a novel SIVsyk antigen-specific Western blot assay to perform a retrospective serosurvey of 276 previously collected serum samples from both wild and captive animals. To develop the antigen-specific Western assay, we propagated SIVsyk strain 173 in a human CEMx174 lymphoid cell line expressing human CCR5. Comparisons between the cross-reactive ELISAs and the SIVsyk Western assay indicate that the antigen-specific Western assay is more specific and sensitive, supporting the utility of using antigen-specific assays in this and other SIV serosurveys. We report that using this Western assay, infection prevalence is higher on average than previously reported, but that the prevalence in wild populations is variable. In captivity and in the wild, females are more commonly infected than males, although these differences are not statistically significant. Positive serology is also common in infants, but no positive juveniles were identified. We interpret this as evidence of maternal antibodies infants, but a low incidence of maternal-fetal transmission.

## Methods

### Specimens

The sera used in this study were obtained between 1981 and 1996 from animals that had been previously trapped in the wild or from previously sampled captive animals housed at the Institute of Primate Research in Nairobi, Kenya. Serum samples were stored continuously at -20°C. All animals were captured, housed and sampled in accordance with humane animal use guidelines established by the Kenya Institute of Primate Research. Wild born monkey samples used in this study were derived from eight locations. In general, these sites were rural or suburban areas that bordered forested areas with natural flowing water. These sites are typical of areas in which humans have encroached on natural habitats and now may come in contact with these monkeys as they forage for food. The geography of the sites varied considerably from heavily forested areas in the highlands of Kenya to drier forests along the lowland coast of Kenya. The range of the lowland Sykes' (*C. mitis kibonotensis *Lonnberg) includes coastal and eastern regions of Kenya [11,13]. The range of the highland Sykes' (*C. mitis kolbi *Neumann) includes the upland central portion of the country [11,13]. The range of the blue Sykes' (*C. mitis stuhlmannii *Matschie) includes western Kenya and the border with Uganda [11,13]. Some specimens from the wild came from unknown locations and these are noted as such. Captive animals used for breeding were housed in an area composed of nine outdoor enclosures. Typically, breeding groups were composed of approximately ten individuals. Each breeding group consisted of a harem of one male and four to six females, plus several infants. Infants were left with mothers for a period of time and were usually moved to another group between the age of six months and two years to prevent inbreeding. Individuals not involved in breeding were kept in groups composed of approximately ten juvenile males and females in larger outdoor enclosures. Animals were aged according to dental inspection and according to sexual maturity. Animals believed capable of mating, usually over two years of age, were considered adults. Infants were less than six months of age.

### ELISA Analysis

Serum samples were tested using the Innotest HIV-1/HIV-2 Antibodies test (Innogenetics, Ghent, Belgium) and Genscreen HIV1/2 version 2 (Biorad, Tokyo, Japan). The Innotest ELISA kit uses synthetic peptides representing HIV-1 subtype M envelope, HIV-2 envelope, and HIV-1 group O envelope antigens. The Genscreen uses purified gp160 envelope and p25 nucleocapsid recombinant protein of HIV-1 and a peptide that mimics the immunodominant epitope of HIV-2 envelope. Each kit was used according to the manufacturers' recommendations. Photometric measurements were performed with a Dynatech MRX plate reader at 450 and 650 nm wavelengths. Positive and negative determinations were based upon cutoff values as determined by the manufacturers' protocols.

### Cells and Virus

CD4+ CEMx174 cells were transduced with a CCR5-encoding vector (pBABE-puro-CCR5, a gift of David Dorsky, University of Connecticut) as previously described [23]. The resulting CCR5 expressing cell line, designated CEM-R5, was maintained in RPMI 1640 with 10% FBS and 1 ug/ml puromycin. The SIVsyk-1.2 isolate (obtained from the AIDS Reference and Reagents Program) was inoculated into CEM-R5 and the CEMx174 parent cell lines. After 7–14 days in culture, widespread syncytia formation was observed in CEM-R5 cells while few syncytia were observed in the parental CEMx174 cells. Cell associated viral proteins were extracted from SIVsyk-1.2 infected CEM-R5 cells grown for 7–10 days post-inoculation.

### Western Blot Analysis

Serum samples were screened by Western blot using SIVsyk-specific viral antigens. SIVsyk-1.2 infected cells were centrifuged for 5 min at 1,000 × g and resuspended in lysis buffer containing 1% triton, 50 mM tris pH 7.5, 2 mM EDTA. Cell debris was pelleted for 2 min. at 12,000 × g and the supernatant was added to loading buffer containing 50 mM Tris-HCl pH 6.8, 100 mM DTT, 2% SDS, 10% glycerol, 0.1% bromophenol blue. Viral antigens were separated on a 12% polyacrylamide gel and subsequently transferred to a PVDF membrane. Membrane strips were blocked with a solution of PBS-T containing 5% dry milk. Serum samples were diluted 1:500 and incubated for one hour at room temperature with individual membrane strips. Primary serum antibodies were detected with a 1:4,000 dilution of goat-anti-rhesus HRP conjugated secondary antibody (Nordic Immunology, Tilburg, Netherlands). Colorimetric detection was performed using the Immunopure Metal Enhanced DAB Substrate Kit (Pierce Endogen, USA).

## Competing interests

The author(s) declare that the have no competing interests.

## Authors' contributions

BRE and EM conducted the ELISA and Western blot experiments and BRE wrote the draft manuscript. DE and JR cultured the SIVsyk virus. MGO provided serum samples and oversaw the ELISA experiments. SFM assisted with and oversaw the Western blot experiments and prepared the final manuscript. All authors read and approved the final manuscript.
